# Glycerol as Alternative Co-Solvent for Water Extraction of Polyphenols from *Carménère* Pomace: Hot Pressurized Liquid Extraction and Computational Chemistry Calculations

**DOI:** 10.3390/biom10030474

**Published:** 2020-03-20

**Authors:** Nils Leander Huamán-Castilla, María Salomé Mariotti-Celis, Maximiliano Martínez-Cifuentes, José Ricardo Pérez-Correa

**Affiliations:** 1 Chemical and Bioprocess Engineering Department, School of Engineering, Pontificia Universidad Católica de Chile, Vicuña Mackenna 4860, P.O. Box 306, Santiago 7820436, Chile; nlhuaman@uc.cl; 2 Escuela de Ingeniería Agroindustrial, Universidad Nacional de Moquegua, Prolongación calle Ancash s/n, Moquegua 18001, Peru; 3 Programa Institucional de Fomento a la Investigación, Desarrollo e Innovación, Universidad Tecnológica Metropolitana, Ignacio Valdivieso 2409, P.O. Box 9845, Santiago 8940577, Chile; 4 Centro Integrativo de Biología y Química Aplicada (CIBQA), Escuela de Tecnología Médica, Facultad de Ciencias de la Salud, Universidad Bernardo O’Higgins, General Gana 1702, Santiago 8370993, Chile

**Keywords:** polyphenols, glycerol, hot pressurized liquid extraction, hydrogen bond, quantum chemical calculations

## Abstract

Glycerol is a co-solvent for water extraction that has been shown to be highly effective for obtaining polyphenol extracts under atmospheric conditions. However, its efficacy under subcritical conditions has not yet been studied. We assessed different water-glycerol mixtures (15%, 32.5%, and 50%) in a hot pressurized liquid extraction system (HPLE: 10 MPa) at 90 °C, 120 °C, and 150 °C to obtain extracts of low molecular weight polyphenols from *Carménère* grape pomace. Under the same extraction conditions, glycerol as a co-solvent achieved significantly higher yields in polyphenols than ethanol. Optimal extraction conditions were 150 °C, with 32.5% glycerol for flavonols and 50% for flavanols, stilbenes, and phenolic acids. Considering gallic acid as a model molecule, computational chemistry calculations were applied to explain some unusual extraction outcomes. Furthermore, glycerol, methanol, ethanol, and ethylene glycol were studied to establish an incipient structure–property relationship. The high extraction yields of gallic acid obtained with water and glycerol solvent mixtures can be explained not only by the additional hydrogen bonds between glycerol and gallic acid as compared with the other alcohols, but also because the third hydroxyl group allows the formation of a three-centered hydrogen bond, which intensifies the strongest glycerol and gallic acid hydrogen bond. The above occurs both in neutral and deprotonated gallic acid. Consequently, glycerol confers to the extraction solvent a higher solvation energy of polyphenols than ethanol.

## 1. Introduction

Chile is the largest world producer of Carménère (~95 million L/year) [1], a highly appreciated wine due to its deep red color and low astringency [2]. These distinctive characteristics are related to its high polyphenol content (240 to 350 mg gallic acid equivalents (GAE)/L) that comes from the skin and seeds of the grapes [2,3]. During winemaking, only a fraction of the skin (25% to 35%) and seed (50% to 70%) polyphenols are extracted [4,5]. Therefore, these solid organic wastes, commonly called grape pomace, represent a not natural source of polyphenols that has not been fully exploited [6]. 

Polyphenols contain an aromatic ring with hydroxyl groups in their chemical structure [5]. These compounds present a diverse range of molecular weights (200 kDa to 3500 kDa) and structural complexity that ranges from rather simple molecules to polymers [3,7]. Skin pomace contains high concentrations of anthocyanins, flavonols, stilbenes, and phenolic acids, while seed pomace is rich in flavanols and phenolic acids [8,9]. These compounds are highly demanded for the production of functional ingredients for the food, nutraceutical, and cosmeceutical industries [10]. 

Grape pomace polyphenols are compounds recognized as GRAS (generally recognized as safe) [11]. Procyanidins, a type of flavanols, have shown antiproliferative activity against breast cancer cells by inducing apoptosis [12]. Furthermore, due to the presence of several hydroxyl groups (OH) in their chemical structure, they also show a strong ability to reduce free radicals (superoxide anion and singlet oxygen) [11]. Flavonols inhibit the release of histamine; therefore, they show anti-inflammatory and anti-allergic bioactivities [11,13]. Phenolic acids inhibit cytochrome P450 bioactivation by various carcinogens and decrease the mutagenicity of sodium azide in Salmonella cells [14,15]. Resveratrol, a stilbene, has shown a multiplicity of activities against several diseases [16,17]. It has been described that resveratrol helps to prevent cancer by scavenging free radicals that induce DNA damage [18,19]. Additionally, it has been found that resveratrol protects against cardiovascular and neurological diseases [20,21,22,23]. In addition, flavanols can modulate astringency in wines, since they interact with saliva proline rich proteins [24] and, recently, they have been shown to be useful to recover soy whey proteins [25]. Phenolic acids present a high thermal resistance and are used as additives in extruded foods [26] and the amphiphilic behavior of flavonols allows their use in encapsulated products [27,28]. Given the wide spectrum of applications of polyphenols, the development and optimization of sustainable technologies to recover them from agroindustrial wastes is a highly active research area [29].

Grape pomace polyphenols are located in the cell vacuoles, which are protected by cellulose, hemicellulose, pectin, and lignin walls [30]. Thus, the extraction method must break these walls in order to release the phenolic compounds [31]. Conventional extraction at atmospheric conditions (1 atm) is one of the most used techniques to recover polyphenols commercially, not only because it is easy to scale up to an industrial level, but also because sustainable processes can be developed using green solvents such as ethanol and glycerol [32,33]. However, conventional techniques such as maceration and Soxhlet extraction require large solvent volumes and long extraction times. Although water-ethanol mixtures are effective to extract polyphenols from different vegetable matrices [34,35,36], water-glycerol mixtures have been shown to be better extractants of polyphenols at atmospheric conditions [37,38,39,40]. Glycerol is a byproduct of the biodiesel industry, recognized as a GRAS low cost solvent [41]. As a co-solvent, glycerol increases the interaction of water with different metabolites of interest, enhancing its solvation ability [33,42,43].

Hot pressurized liquid extraction (HPLE) is a sustainable technology for the recovery of non-polar and polar substances [44]. This technology uses solvents at high temperatures and elevated pressures (temperature 100 °C to 250 °C; and pressure 0.1 to 22 MPa), keeping the solvent in a liquid state during the whole extraction procedure, enhancing the solubility and mass transfer of the analytes [45]. However, high water temperatures (≥120 °C) significantly affect the chemical composition of the extracts due to polyphenol hydrolysis and formation of toxic compounds (Maillard compounds such as hydroxymethylfurfural, HMF) [46,47]. Co-solvents such as ethanol reduce the solvent polarity which can explain the significant improvement of polyphenols recovery and the considerable reduction in sugar extraction and HMF formation [48,49,50,51,52]. Glycerol, however, has been shown to be a better co-solvent than ethanol to recover polyphenols in conventional extractions; as far as we are aware there is no reported application of water-glycerol mixtures in HPLE. 

Empirical methods such as Kamlet–Taft parameters have been applied to assess the ability of different solvents, such as ethanol and glycerol, to form hydrogen bonds with a given solute [44]. This ability is defined by the α parameter, although its measurement is inaccurate [42,43]. Specific interaction values for intra- and intermolecular hydrogen bonds can be estimated more accurately with alternative tools such as electronic structure calculations [53]. Natural bond orbital (NBO) analysis has been one of the most used electronic structure methods to characterize hydrogen bonds [54,55,56]. This involves population analysis and distributing electron density into orbitals, allowing us to perform quantitative bonding analysis similar to those in traditional chemistry [57]. The interaction between filled (bonding) and unoccupied (antibonding) orbitals is used as a measure of delocalization (stabilization energy) due to the presence, for instance, of hydrogen bond interactions. This approach seems appropriate for an improved understanding of specific cases such as interactions between solvents (i.e., ethanol and glycerol) and a given polyphenol.

In this study we assessed the effect of glycerol concentrations (15% to 50%) and high temperatures (90 °C to 150 °C) on the recovery of specific low molecular weight polyphenols from *Carménère* pomace using HPLE. Additionally, computational calculations were carried out to unveil why glycerol is such a good co-solvent to recover polyphenols.

## 2. Materials and Methods 

### 2.1. Grape Pomace

*Carménère* pomace was provided by Concha y Toro vineyard from Chile. The samples were stored at –20 °C until analysis. Then, frozen samples were ground to ~2 mm using a cutting mill Oster blender (Sunbeam Products, Inc., Boca Raton, FL, USA).

### 2.2. Chemicals and Analytic Reagents

Glycerol (≥98%) was provided by Sigma-Aldrich Chemical Co. (St. Louis, MO, USA). Phenolic standards included: catechin (≥98%), epigallocatechin (≥98%), epicatechin (≥98%), kaempferol (≥98%), resveratrol (≥98%), quercetin (≥97%), gallic acid (≥99%), caffeic acid (≥99%), chlorogenic acid (≥98%), vanillic acid (≥99%), protocatechuic acid (≥98%), and ferulic acid (≥98%), and were provided from Xi’an Haoxuan Bio-Tech Co., Ltd. (Baqiao, China).

### 2.3. Hot Pressurized Liquid Extraction (HPLE) of Carménère Pomace

Polyphenol extracts were obtained from *Carménère* pomace using water-glycerol mixtures (15%, 32.5%, and 50%) at 90 °C, 120 °C, and 150 °C in a pressurized extraction device (ASE 150, Dionex, Sunnyvale, CA, USA). The experimental range of study, both for temperature and co-solvent level, was defined based on previous research [52,53] and prelaminar tests. The highest HPLE temperature (150 °C) was established considering the occurrence of HMF in the extracts. At this temperature, for all co-solvent addition levels, HMF was not detected. Glycerol concentrations higher than 50% did not allowed us to control the extraction process and the subsequent stabilization of the extracts. Five grams of sample (dry weight, dw) was mixed with 40 g of quartz sand and placed in a 100 mL extraction cell. The extraction parameters were as follows: extraction solvent volume 50 mL, pressure 10 MPa, one extraction cycle, 100 mL of washing volume, 250 s of nitrogen purge time, and 5 min of static extraction time. After extraction, the crude extracts were collected in amber vials and frozen at −20 °C until chemical analysis. Extraction and analysis were performed in triplicate with the data presented as mean and coefficient of variation (CV).

### 2.4. Quantification of Specific Polyphenols

Specific families of polyphenols were quantified using a ultra-performance liquid chromatography tandem mass spectrometry (UPLC-MS) (Dionex Ultimate 3000 with Detector MS Orbitrap Exactive plus, Thermofisher, Waltham, MA, USA) equipped with a reverse phase Acquity UPLC BEH C18 column (1.7 µm × 2.1 × 100 mm) according to the methodologies of Liu et al. [58] and Huaman-Castilla et al. [53]. Polyphenols were quantified in the range 0.05–1.5 µg/L (limit of detection (LOD) 0.03 and limit of quantification (LOQ) 0.05). Absolute calibration curves were constructed for each polyphenol, presenting in all cases a good linearity (r^2^ = 0.999). The method also presented an acceptable precision for all determined compounds, with relative standard deviations (RSD%) of ±10%. Analyses were all carried out in triplicate and results were expressed as µg of specific polyphenols per gram of dry weight of grape pomace (gdp).

### 2.5. Statistical Analysis

A factorial experimental design was applied to determine the effect of extraction temperature and co-solvent during extraction on the global recovery of specific polyphenols which followed the methodology proposed by Mariotti-Celis et al. [52]. In addition, mean and CV results were presented. Analysis of variance (ANOVA) and least significant difference tests were applied to the response variables (*p* ≤ 0.05). The statistical analyses of data were carried out using the software Statgraphics Plus for Windows 4.0 (Statpoint Technologies, Inc., Warrenton, VA, USA). 

### 2.6. Computational Chemistry Calculations

Geometrical optimization was carried out using the Gaussian 09 software [59], at density functional theory (DFT) M06-2X/6-311+G(d,p) level of theory. No imaginary vibrational frequencies were found at the optimized geometries, indicating that they are the true minimal of the potential energy surface. Then, NBO analysis was carried out employing NBOPro 6.0 software to calculate the donor acceptor interactions (stabilization energy) due to the intra- and intermolecular hydrogen bonds present in gallic acid, methanol, ethanol, ethylene glycol, and glycerol.

## 3. Results and Discussions

### 3.1. Use of Glycerol as Alternative Co-Solvent in Hot Pressurized Liquid Extraction 

#### 3.1.1. Phenolic Acids Extraction

**Figure 1:**
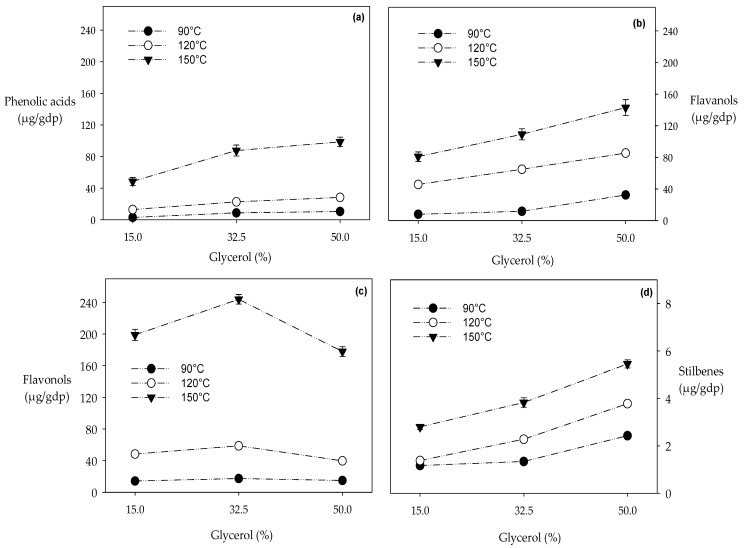
Effect of glycerol as a co-solvent during hot pressurized liquid extraction system (HPLE) to obtain extracts rich in (**a**) phenolic acids; (**b**) flavonols; (**c**) flavanols; and (**d**) stilbenes.

A 50% water-glycerol mixture at 150 °C increased the extractability of phenolic acids from 2.87 µg/gdp (15%, 90 °C) to 98.53 µg/gdp (Table 1 and Figure 1a). A similar behavior was observed for ethanol-water mixtures which achieved maximum recovery of phenolic acids in HPLE at 150 °C and 50% of co-solvent, although with a lower yield (~85 µg/gdp) [53]. Five phenolic acids were quantified in the HPLE extracts, where the gallic acid content was significantly higher (~71 µg/gdp) than the others. At the highest glycerol concentration (50%), the highest HPLE temperature (150 °C) recovered ~13 times more gallic acid than the lowest (90 °C). However, moving from 32.5% to 50% of glycerol has almost no impact. *Carménère* grape pomace presents high amounts of gallic acid, which is galloylated with procyanidins (flavanols) [2,3]. Therefore, high temperatures (≥120 °C) promote the hydrolysis of the galloylated compounds [60], releasing free gallic acid in large quantities.

#### 3.1.2. Flavanols Extraction

Similar to phenolic acids, the extraction of flavanols was significantly enhanced with increasing amounts of glycerol in the solvent and with increasing extraction temperatures: from 7.94 µg/gdp (15%, 90 °C) up to 143.03 µg/gdp (50%, 150 °C) (Table 1 and Figure 1b). Previous studies with HPLE showed that with ethanol as the co-solvent, much higher recoveries of flavanols were obtained. Monrad et al. [51] reported that water-ethanol mixtures (30%) at 140 °C recovered ~58% more flavanols than pure water at 140 °C. Extracting with HPLE and with the same pomace that we used here, Huaman-Castilla et al. [53] achieved maximum recovery of flavanols (~105.46 µg/gdp) with 32.5% ethanol and 150 °C, that is ~26% less than what we recovered with 50% glycerol at the same temperature. Under all extraction conditions, more epigallocatechin was recovered than catechin and epicatechin (the other flavanols quantified in this study). This trend was also observed with HPLE processing of *Carménère* pomace using ethanol-water mixtures [53]. Epigallocatechin is the most abundant flavanol in *Carménère* skin [3], and has six hydroxyl groups, while catechin and epicatechin have only five [61]; therefore, epigallocatechin interacts more with these extraction solvents.

#### 3.1.3. Flavonols Extraction

Flavonols extraction was optimal at 32.5% glycerol and 150 °C, reaching much higher levels (143.58 µg/gdp) than extractions at 15% glycerol and 90 °C (14.21 µg/gdp) (Table 1 and Figure 1c). At all extraction temperatures, a glycerol concentration of 50% significantly decreased (~30%) the recovery of the flavanols quantified in this study (quercetin and kaempferol). Previous studies have reported that flavonols were more soluble at low ethanol concentrations (15%). Wijngaard et al. [62] found that an increase from 15% to 50% of ethanol at 100 °C decreased ~40% the recovery of flavonols expressed as rutin. Huaman-Castilla et al. [53] observed in HPLE processing a reduction of ~42% in the recovery of flavonols when ethanol concentrations increased from 15% to 50% at 150 °C. Flavonols are relatively polar due to the presence of carbonyl and hydroxyl groups in their chemical structure; hence, it is expected that relatively polar solvent mixtures should perform better than nonpolar solvent mixtures. In this study, we found that optimal HPLE/glycerol-water processing (32.5%, 150 °C) recovered ~4 times more flavonols than optimal HPLE/ethanol-water processing (15%, 150 °C) [53]. The carbonyl and hydroxyl groups in the flavanols’ chemical structure could easily form hydrogen bonds with the functional groups of glycerol [63]. Under almost all extraction conditions, 10 times more quercetin was recovered than kaempferol. In *Carménère* skin, quercetin is much more abundant than kaempferol and quercetin presents more hydroxyl groups than kaempferol.

#### 3.1.4. Resveratrol Extraction

Like phenolic acids and flavanols, resveratrol recovery increased with the amount of glycerol in the solvent and with the extraction temperature, i.e., from 1.17 µg/gdp (15%, 90 °C) to 5.45 µg/gdp (50%, 150 °C) (Table 1 and Figure 1d). In previous HPLE research [53], we found that the optimal resveratrol extraction was at the same temperature (150 °C) but at an intermediate ethanol concentration (32.5%), although less resveratrol was obtained (~4.28 µg/gdp). The ability of glycerol as a co-solvent to improve polyphenols extraction as compared with other solvents such as ethanol was also reported by Manousaki et al. [33].

### 3.2. Quantum Chemical Calculations

To get insight into the effect of the solvent mixture on the molecular solvation of polyphenols, we carried out DFT quantum chemical calculations. M06-2X functional was employed together with the 6-311+G(d,p) basis set. This functional is a hybrid with a 54% Hartree Fock (HF) exchange, which has been successfully applied to free solvation energies of polyphenols radical scavengers [64,65] to evaluate local acid strength [66] and to study resonance assisted intramolecular hydrogen bonds [67]. Among the polyphenols quantified in this study, gallic acid was taken as a model molecule. In previous work, we theoretically studied the solvation of a series of polyphenols present in water-ethanol HPLE extracts from *Carménère* pomace, including gallic acid [53]. In this work, glycerol as a water co-solvent achieved higher polyphenol yields than ethanol as a water co-solvent, at the same extraction conditions. It is reasonable to suggest that glycerol confers better solvation properties than ethanol to the solvent mixture; however, it is not clear how this occurs from a molecular point of view.

To establish a preliminary structure–property relationship between the co-solvent (alcohol) and the solubility of gallic acid in the extraction solvent (water-alcohol mixture), we analyzed the water-alcohol-gallic acid complexes for methanol, ethanol, ethylene glycol, and glycerol. Both neutral and anionic (deprotonated) species are studied, since there is no experimental information about the pKa of the acid group of gallic acid in theses mixtures. Until now, the only studies that had determined the pKas of gallic acid in mixtures of water with organic solvent were from Beltran et al. [68] and from Jabbari [69], where they studied water-acetonitrile mixtures. In the first work, it was found that the first pKa of the gallic acid changed from 4.27 to 5.18 when the solvent changed from 100% water to water/acetonitrile 70:30. In the second work, it was found that the first pKa of the gallic acid changed from 4.39 to 6.82 when the solvent changed from 100% to water/acetonitrile 10:90. On the basis of the above, it is reasonable to speculate that for mixtures 50:50 water/alcohol, the pKa of the acid group could reach values around six. This analysis allowed us to define how intermolecular hydrogen bonds change as the number of carbons and hydroxyls in the co-solvent changes. Although methanol and ethylene glycol are not food grade solvents, their inclusion helped us to establish the structure–property relationship. 

**Figure 2:**
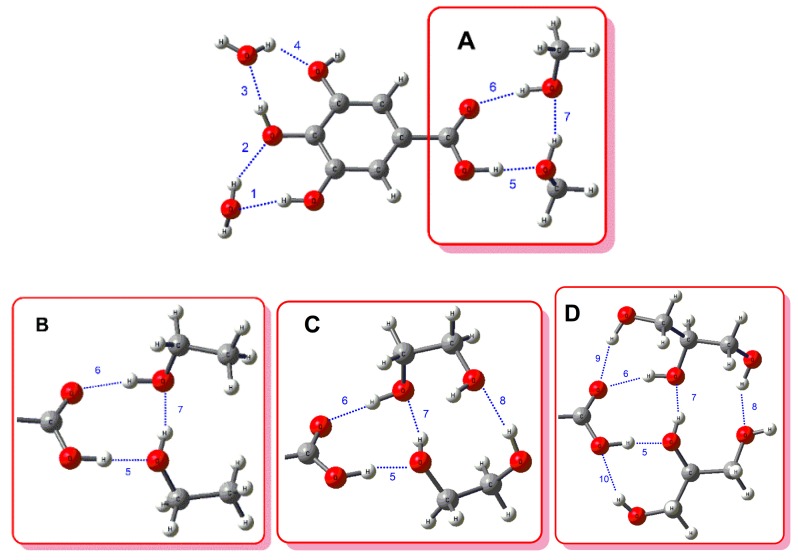
Optimized structures at density functional theory (DFT) M06-2X/6-311+G(d,p) level for gallic acid and water with methanol (**A**); ethanol (**B**); ethylene glycol (**C**); and glycerol (**D**).

The complexes for neutral gallic acid with water and alcohol mixtures were optimized considering the formation of intermolecular hydrogen bonds between two water molecules and two alcohol molecules with one gallic acid molecule. This configuration was selected because the carboxylic acid group in gallic acid has both donor and acceptor hydrogen bond capabilities, therefore, this configuration was more suitable to assess the effects of alcohol’s structural changes on the strength of the carboxylic acid hydrogen bonds. Figure 2 shows the optimized geometry for gallic acid and water with methanol (A); ethanol (B); ethylene glycol (C); and glycerol (D). Table 2 shows hydrogen bond distances, and stabilization energies (ΔE_ij_^(2)^) for water-alcohol-gallic acid complexes (A, methanol; B, ethanol; C, ethylene glycol: D, glycerol). On the one hand, the hydrogen bonds between water molecules and gallic acid (numbered as 1, 2, 3, and 4 in the Figure 2) were common to the four complexes and presented similar distances and stabilization energies; their values are given in the Supplementary Materials.

On the other hand, hydrogen bonds between gallic acid and alcohols showed remarkable differences in parameter values depending on the alcohol structure. Carboxylic acid-hydroxyl hydrogen bonds (5, 6) and hydroxyl-hydroxyl hydrogen bond (7) were common to the four alcohols. Ethylene glycol and glycerol showed an additional hydroxyl-hydroxyl hydrogen bond (8), while glycerol presented two additional carboxylic acid-hydroxyl hydrogen bonds (9, 10). As expected, hydrogen bond distances are inversely proportional to the stabilization energies (ΔE_ij_^(2)^). Hydrogen bond 5, where the carboxylic acid acted as a donor and one hydroxyl of the alcohols acted as an acceptor, presented in all cases the shortest distance and the highest stabilization energy (ΔE_ij_^(2)^). As the size of the alcohols increased from methanol to ethylene glycol, the stabilization energy of bond 5 decreased. Glycerol breaks this trend with an unexpectedly large increment in this stabilization energy. For ethanol and ethylene glycol, the reduction in the stabilization energy of 5 was compensated by an increment in the other hydrogen bond energies; therefore, the sum (∑) of them increased from methanol to ethylene glycol. Although the additional carboxylic acid-hydroxyl bonds in glycerol (9 and 10) were not strong, they notably influenced bonds 5 and 6, making the first much stronger and the second much weaker. Overall, considering the energy changes in the common bonds (5, 6, 7) and the additional bonds (8, 9, 10), the sum of the hydrogen bond stabilization energies of glycerol is the highest.

The optimization for deprotonated gallic acid was attempted considering a similar started conformation than for neutral, with two water molecules solvating to phenol groups and two alcohol solvating to acid group, but it was no possible to find a minimum. Hence, we modified the starting conformation, putting one water and one alcohol molecule to solvate the phenolic groups and the same for the solvation of anion carboxylate group. Starting from this conformation, we found optimized structures for the four cases.

**Figure 3:**
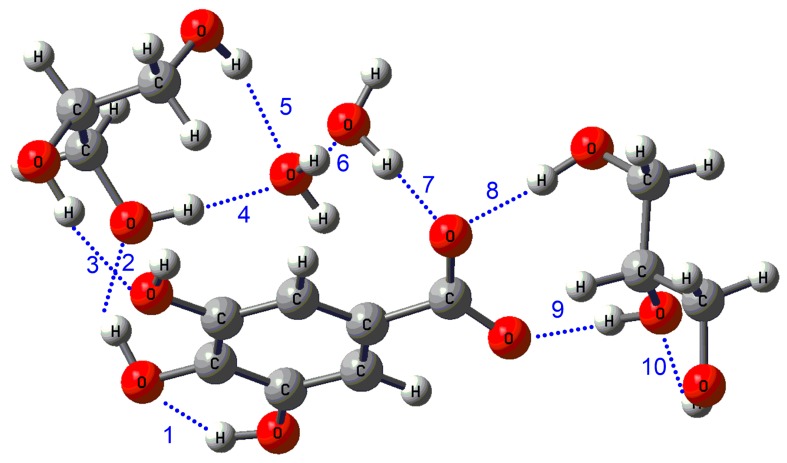
Optimized structures at DFT M06-2X/6-311+G(d,p) level for deprotonated gallic acid with glycerol and water.

Figure 3 shows the optimized geometry for deprotonated gallic acid solvated by water with glycerol. Table 3 shows the values of the hydrogen bond distances and stabilization energies (ΔEij(2)) for deprotonated gallic acid solvated by water and glycerol. The detailed data for the gallic acid solvated by water-methanol, water-ethanol, and water-ethylene glycol can be found in the Supplementary Materials.

Table 4 summarizes the values for the sum of the stabilization energies (ΔE_ij_^(2)^) of the hydrogen bonds for the four cases. The sum of the stabilization energies for all hydrogen bonds (∑Total) showed that it diminishes when methanol changed to ethanol (74.21 to 70.88 kcal, respectively), but specific hydrogen bonds with the carboxylate anion presented almost the same value (42.88 to 42.66 kcal). When the alcohol changes to ethylene glycol, a notable increase of the ∑Total occurred to reach 85.84 kcal, while specific hydrogen bonds with carboxylate anion increased slightly in mixtures with methanol and ethanol. The above indicates that the increase in the ∑Total from methanol and ethanol to ethylene glycol is mainly due to the solvation of the phenolic groups. Finally, the ∑Total in water-glycerol increased significantly to 127.93 kcal, and the sum for the hydrogen bonds with the carboxylate group (∑carboxylate group) also increased significantly to 60.14 kcal. The latter indicates that the presence of the third hydroxyl group in glycerol has a much greater impact than expected considering the trend followed by the other alcohols.

These results show that either for neutral or deprotonated species of gallic acid, glycerol presents much stronger hydrogen bonds than the other alcohols, resulting in a better solvation capacity. The above can explain why glycerol is a more effective co-solvent than ethanol to recover polyphenols. 

## 4. Conclusions

We developed a sustainable HPLE process using glycerol as an alternative green and low-cost co-solvent for the recovery of specific families of polyphenols from *Carménère* grape pomace at high temperatures. The highest recoveries were obtained at 150 °C with 50% of glycerol for phenolic acids, flavanols, and stilbenes, and with 32.5% of glycerol for flavonols. A preliminary structure–property relationship was established applying quantum chemical calculations, using gallic acid, both in neutral and deprotonated form, as a model polyphenol and methanol, ethanol, ethylene glycol, and glycerol as co-solvents. It was found that in the neutral form the strongest interaction between gallic acid and the studied alcohols was the hydrogen bond between the carboxylic acid group in gallic acid (acting as a donor) and a hydroxyl group in the alcohols (acting as an acceptor). Glycerol did not follow the decreasing trend in stabilization energy of this bond with the molecular weight of the alcohols. The third hydroxyl group in glycerol allowed cooperative multiple hydrogen bonds, influencing the stabilization energies of the common hydrogen bonds and increasing the sum of stabilization energies. In deprotonated form of gallic acid, we found that the strongest interaction with the studied alcohols was the hydrogen bonds between the carboxylate anion group in gallic acid (acting as an acceptor) and water and alcohols (acting as a donors). As was the case with neutral species, glycerol presented hydrogen bonds notably stronger than the other alcohols, indicating that the presence of the third hydroxyl group in glycerol has a much greater impact than expected considering the trend followed by the other alcohols. Therefore, glycerol confers better solvation properties to the solvent mixture than ethanol during the HPLE of polyphenols from *Carménère* grape pomace at high temperatures.

## Figures and Tables

**Table 1:** Specific polyphenols quantified in the extracts obtained by HPLE.

Description	Temperature (°C)								
	90 °C			120 °C			150 °C		
	Glycerol (%)			Glycerol (%)			Glycerol (%)		
	15%	32.5%	50%	15%	32.5%	50%	15%	32.5%	50%
**Acids (µg/gdp)**	Mean CV	Mean CV	Mean CV	Mean CV	Mean CV	Mean CV	Mean CV	Mean CV	Mean CV
Gallic	1.99a 0.06	4.53b 0.09	5.54b 0.08	7.42a 0.11	15.35b 0.09	19.69c 0.11	37.48a 0.11	67.86b 0.08	70.84c 0.10
Chlorogenic	0.88a 0.07	0.93a 0.08	0.45b 0.06	1.03 a 0.04	0.85a 0.05	0.17a 0.09	2.87a 0.12	3.04a 0.11	2.25a 0.07
Vanillic	ND	2.37 a 0.03	3.31a 0.07	2.91a 0.06	4.37a 0.08	7.40b 0.02	5.14a 0.04	12.75b 0.09	19.15c 0.05
Caffeic	ND	0.81a 0.10	0.93a 0.11	0.91a 0.10	1.14a 0.10	0.68a 0.12	1.28a 0.09	3.39b 0.09	5.32c 0.07
Ferulic	ND	ND	0.14 0.08	0.56a 0.03	0.86a 0.06	0.29a 0.09	0.65a 0.05	0.52a 0.11	0.97a 0.13
**Σ:**	**2.87 a 0.08**	**8.64 b 0.08**	**10.37b 0.07**	**12.83a 0.08**	**22.57b 0.08**	**28.21c 0.08**	**48.42a 0.09**	**87.56b 0.10**	**98.53c 0.09**
**Flavanols (µg/gdp)**									
Catechin	1.79a 0.06	3.63b 0.06	12.14c 0.09	9.25a 0.09	15.19b 0.11	25.20c 0.09	22.43a 0.05	29.06b 0.08	39.26c 0.11
Epicatechin	1.71a 0.09	3.21b 0.08	8.50c 0.07	10.35a 0.06	14.75b 0.08	16.50b 0.11	13.15 0.05	22.17 0.04	19.92 0.09
Epigallocatechin	4.44a 0.05	4.85a 0.04	11.75b 0.06	26.11a 0.10	34.96b 0.08	43.72c 0.12	45.34a 0.11	57.94b 0.12	83.85c 0.10
**Σ:**	**7.94 a 0.07**	**11.70b 0.06**	**32. 39c 0.07**	**45.71a 0.09**	**64.90b 0.09**	**85.42c 0.11**	**80.92a 0.06**	**109.17b 0.08**	**143.03c 0.10**
**Flavonols (µg/gdp)**									
Quercetin	12.28a 0.04	14.53b 0.06	13.46a,b 0.07	39.77a 0.10	47.26b 0.09	35.53c 0.11	184.35a 0.06	257.60b 0.12	166.95c 0.11
Kaempherol	1.93a 0.06	2.79a 0.06	1.41a 0.03	8.58a 0.09	11.36b 0.13	4.24c 0.06	14.58a 0.09	26.36b 0.13	11.24c 0.06
**Σ:**	**14.21a 0.05**	**17.32b 0.06**	**14.87a 0.04**	**48.35a 0.09**	**58.62b 0.11**	**39.77c 0.08**	**198.93a 0.08**	**243.96b 0.13**	**177.89c 0.08**
**Stilbenes (µg/gdp)**									
Resveratrol	1.17a 0.08	2.34a 0.06	2.43a 0.04	1.38a 0.06	2.18a,b 0.08	3.78b 0.09	2.80a 0.08	3.83a 0.09	5.45b 0.06

CV, coefficient of variation; ND, not detected; different letters (a, b, c) mean significant differences (p = 0.05) between concentrations of each polyphenol at the HPLE conditions studied.

**Table 2:** Calculated distances, and stabilization energies for the hydrogen bonds between gallic acid, water and methanol (**A**); ethanol (**B**); ethylene glycol (**C**); and glycerol (**D**).

	Hydrogen Bond Distances (Å)						Hydrogen Bond ΔE_ij_^(2)^ (kcal)						
Alcohol	5	6	7	8	9	10	5	6	7	8	9	10	∑
A	1.59	1.81	1.76	−	−	−	38.73	13.90	17.57	−	−	−	70.20
B	1.60	1.84	1.72	−	−	−	36.77	11.95	22.75	−	−	−	71.47
C	1.63	1.84	1.79	1.88	−	−	33.76	12.03	18.07	9.54	−	−	73.40
D	1.55	2.07	1.74	2.07	1.89	2.12	44.58	3.46	20.31	2.44	5.76	2.95	79.50

**Table 3:** Calculated distances, and stabilization energies at DFT M06-2X/6-311+G(d,p) level for the hydrogen bonds between deprotonated gallic acid, glycerol and water.

Hydrogen Bond (HB)	Distances (Å)	ΔE_ij_^(2)^ (kcal)
**1**	2.11	2.48
**2**	1.84	11.55
**3**	2.04	5.24
**4**	1.79	17.94
**5**	2.04	3.52
**6**	1.70	24.10
**7**	1.64	23.54
**8**	1.78	16.17
**9**	1.77	20.43
**10**	2.10	2.96
		∑=127.93

**Table 4:** Sum of the stabilization energies for all hydrogen bonds (∑_Total_) and for the hydrogen bonds with the carboxylate group (∑_carboxylate group_) of the deprotonated gallic acid in the four solvent mixtures (1:1 water/alcohol).

Solvent Mixture	∑_Total_ (kcal)	∑_carboxylate group_ (kcal)
**water-methanol**	74.21	42.88
**water-ethanol**	70.88	42.66
**water-ethylene glycol**	85.84	43.33
**water-glycerol**	127.93	60.14

## Supplementary Materials

The following are available online at https://www.mdpi.com/2218-273X/10/3/474/s1, Table S1: Calculated distances and stabilization energies for the hydrogen bonds between gallic acid and methanol (A), ethanol (B), ethylene glycol (C) and glycerol (D); Table S2: Calculated distances and stabilization energies for the hydrogen bonds between deprotonated gallic acid and methanol; Table S3: Calculated distances and stabilization energies for the hydrogen bonds between deprotonated gallic acid and ethanol; Table S4: Calculated distances and stabilization energies for the hydrogen bonds between deprotonated gallic acid and ethylene glycol; Cartesian coordinates for all the calculated optimized structures.
