# Supplementary material for: Glycerol as Alternative Co-Solvent for Water Extraction of Polyphenols from Carménère Pomace: Hot Pressurized Liquid Extraction and Computational Chemistry Calculations
Source: Biomolecules. 2020 Mar 20;10(3):474. doi: 10.3390/biom10030474 (PMC7175273; doi:10.3390/biom10030474)
Supplement: Supplementary file 1 [file biomolecules-10-00474-s001.pdf]

Supplementary Information

Glycerol as Alternative Co-Solvent for Water Extraction of Polyphenols from *Carménère* Pomace: Hot Pressurized Liquid Extraction and Computational Chemistry Calculations

Nils Leander Huamán-Castilla [1,4], María Salomé Mariotti-Celis [2,*], Maximiliano Martínez-Cifuentes [3,*] and José Ricardo Pérez-Correa [1,*]

1 Chemical and Bioprocess Engineering Department, School of Engineering, Pontificia Universidad Católica de Chile, Vicuña Mackenna 4860, P.O. Box 306, Santiago 7820436, Chile.
2 Programa Institucional de Fomento a la Investigación, Desarrollo e Innovación, Universidad Tecnológica Metropolitana, Ignacio Valdivieso 2409, P.O. Box 9845, Santiago 8940577, Chile.
3 Centro Integrativo de Biología y Química Aplicada (CIBQA), Escuela de Tecnología Médica, Facultad de Ciencias de la Salud, Universidad Bernardo O'Higgins, General Gana 1702, Santiago 8370993, Chile.
4 Escuela de Ingeniería Agroindustrial, Universidad Nacional de Moquegua, Prolongación calle Ancash s/n, Moquegua 18001, Perú.

* Corresponding authors: mmariotti@utem.cl (M.S. Mariotti-Celis); maximiliano.martinez@ubo.cl (M. Martínez-Cifuentes); perez@ing.puc.cl (J.R. Pérez-Correa)

Table S1. Calculated distances and stabilization energies for the hydrogen bonds between gallic acid and methanol (A), ethanol (B), ethylene glycol (C) and glycerol (D)

	**HB Distances (Å)**				**HB $\Delta E_{ij}^{(2)}$ (kcal)**			
**Alcohol**	**1**	**2**	**3**	**4**	**1**	**2**	**3**	**4**
**A**	1.84	1.97	1.74	2.00	13.31	4.10	19.49	4.12
**B**	1.84	1.97	1.74	2.00	13.27	4.15	19.45	4.12
**C**	1.84	1.98	1.73	2.01	13.60	4.21	19.75	3.84
**D**	1.83	1.99	1.73	2.03	14.21	4.00	20.09	3.47

A: water-methanol; **B**: water-ethanol; **C**: water-ethylene glycol; **D**: water glycerol

Table S2. Calculated distances and stabilization energies for the hydrogen bonds between deprotonated gallic acid and methanol.

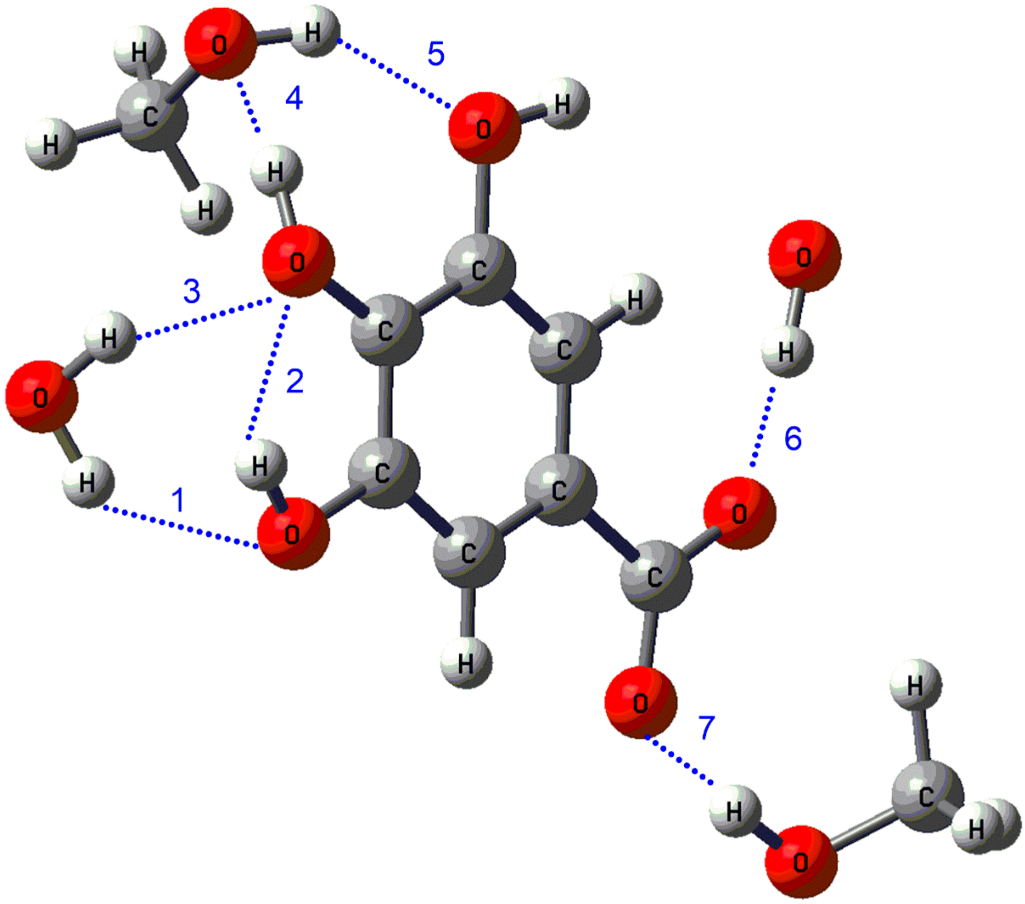

Hydrogen bonding	HB Distances (Å)	HB $\Delta E_{ij}^{(2)}$ (kcal)
**1**	2.38	0.81
**2**	2.16	1.08
**3**	2.08	4.45
**4**	1.77	17.83
**5**	1.92	7.16
**6**	1.70	23.38
**7**	1.77	19.50
		$\sum$ = 127.93

Table S3. Calculated distances and stabilization energies for the hydrogen bonds between deprotonated gallic acid and ethanol.

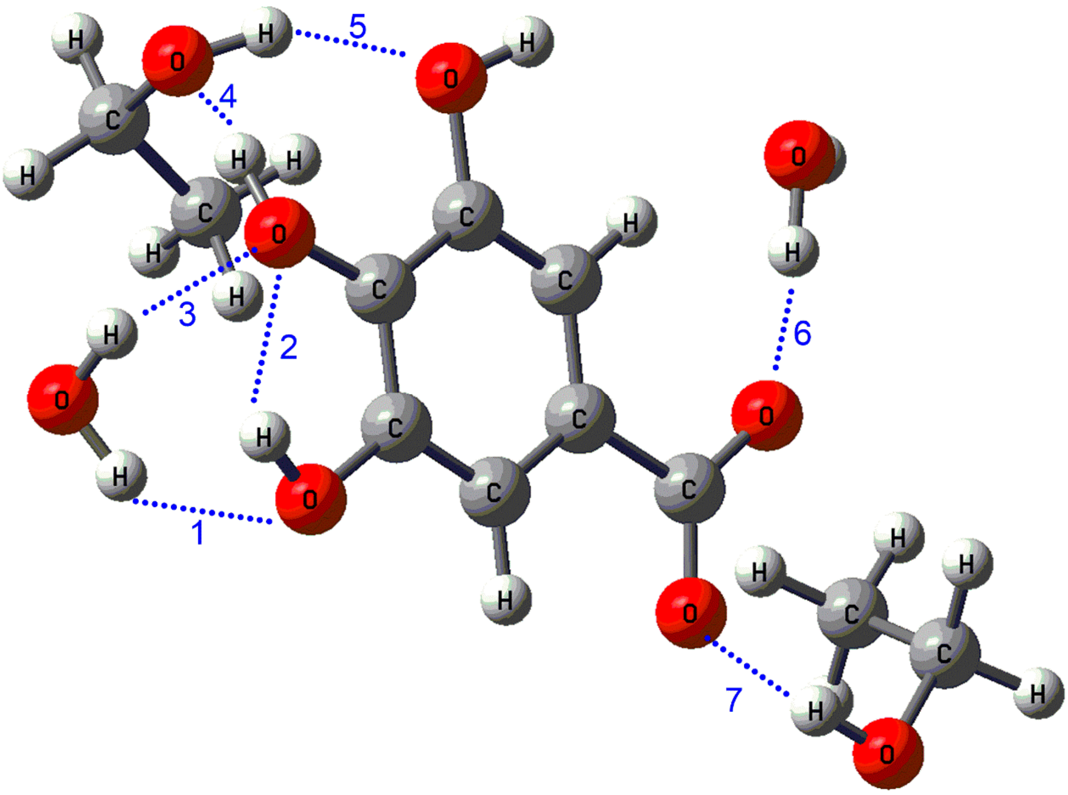

Hydrogen bonding	HB Distances (Å)	HB $\Delta E_{ij}^{(2)}$ (kcal)
**1**	2.37	0.86
**2**	2.16	1.08
**3**	2.11	3.89
**4**	1.76	18.90
**5**	1.96	3.49
**6**	1.70	23.29
**7**	1.78	19.37
		$\sum$ = 127.93

Table S4. Calculated distances and stabilization energies for the hydrogen bonds between deprotonated gallic acid and ethylene glycol.

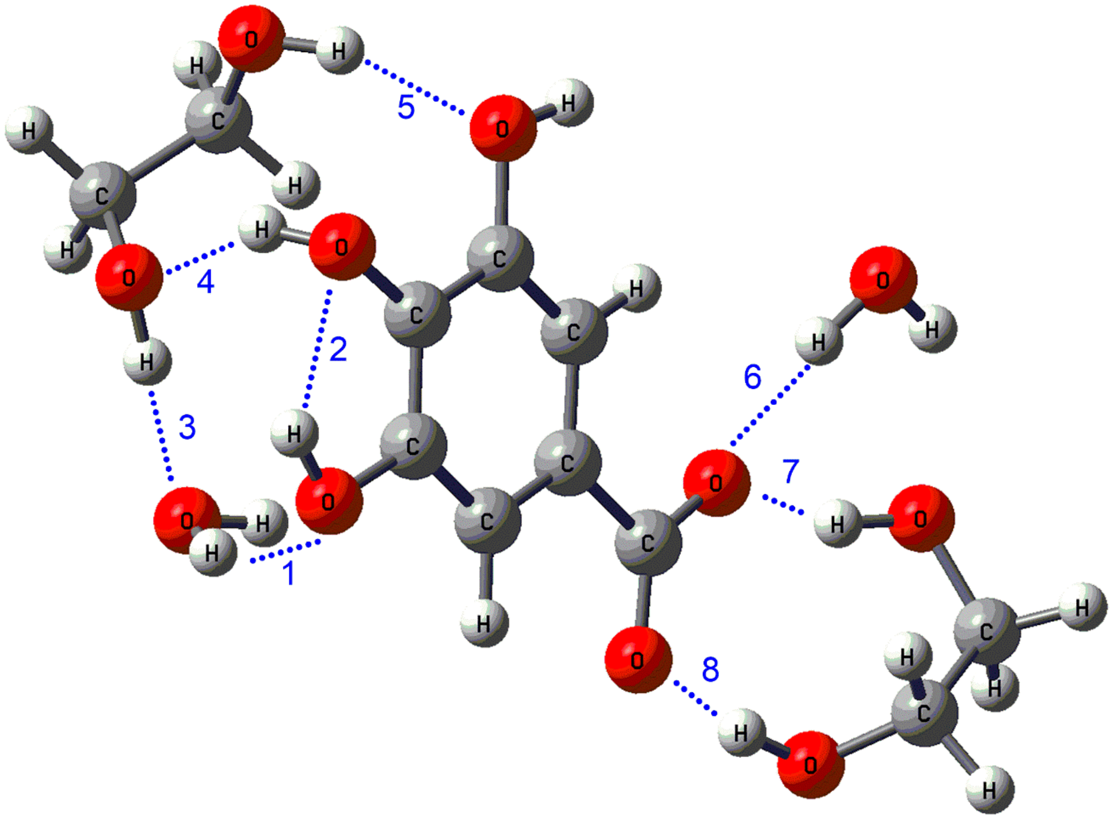

Hydrogen bonding	HB Distances (Å)	HB $\Delta E_{ij}^{(2)}$ (kcal)
**1**	2.12	2.93
**2**	2.13	2.65
**3**	1.82	15.83
**4**	1.86	13.22
**5**	1.92	7.88
**6**	1.85	8.40
**7**	1.83	14.64
**8**	1.77	20.29
		∑ = 127.93

Optimized geometries

M062X/6-311+G(d,p) level

Neutral gallic acid

Water-methanol (A)

C	-0.63584000	-1.23551800	0.39034000
C	-2.02486900	-1.13683200	0.35665400
C	-2.62654900	0.04793000	-0.09244100
C	-1.81707200	1.10724800	-0.51210200
C	-0.43788400	1.01635600	-0.46645600
C	0.14855200	-0.16491800	-0.01735100
H	-0.18761900	-2.15410700	0.74417700
H	0.19252200	1.84158800	-0.77866200
O	-2.74226800	-2.19475900	0.77099100
H	-3.69634700	-2.10664000	0.59222400
O	-3.98719300	0.10690300	-0.14465600
H	-4.31803700	0.98994800	0.12000700
O	-2.45548700	2.24474800	-0.96248600
H	-1.81024500	2.80018600	-1.40884000
C	1.63452800	-0.23793200	0.01162600
O	2.10817700	-1.39219900	0.42239300
H	3.11494700	-1.39982400	0.39864900
O	2.33460800	0.70485900	-0.32431700
O	4.69674100	-1.46058900	0.21143200
H	5.02752700	-0.54842800	0.32876600
O	-4.67187000	2.62103700	0.60336500
H	-3.99103000	3.02506000	0.05080900
H	-5.48001800	3.12471400	0.48965700
O	-5.41719000	-2.21358700	-0.05853300
H	-5.66126800	-2.81290800	-0.76645100
H	-5.28547400	-1.34305600	-0.45502100
O	5.01771400	1.19509400	0.07389300
H	4.06701000	1.19242800	-0.13383100
C	5.32006000	2.27741700	0.93487800
H	4.72006600	2.24515100	1.84940300
H	5.15800200	3.23657700	0.43491900

C	5.01293200	-1.87383600	-1.11270700
H	4.56049700	-2.85285500	-1.26770400
H	4.61353200	-1.17120000	-1.85014200
H	6.37295900	2.20016700	1.20386000
H	6.09398000	-1.95834400	-1.24313400

Water-ethanol (B)

C	-1.31377100	-1.20457000	0.33564600
C	-2.70571100	-1.15676600	0.34772300
C	-3.36464400	0.00676400	-0.07575900
C	-2.60874400	1.09610000	-0.51760100
C	-1.22632600	1.05531100	-0.51792600
C	-0.58283300	-0.10492900	-0.09342300
H	-0.82004800	-2.10709600	0.66950500
H	-0.63652400	1.90418500	-0.84591900
O	-3.37061100	-2.24133400	0.78140300
H	-4.33069000	-2.19279500	0.62066600
O	-4.72791900	0.01642500	-0.08181900
H	-5.08068300	0.88442000	0.20332200
O	-3.30254900	2.21108400	-0.94151800
H	-2.69248900	2.79152600	-1.40523700
C	0.90572600	-0.12378400	-0.11393500
O	1.43164800	-1.26184500	0.27907000
H	2.43506300	-1.22870000	0.25078800
O	1.55994200	0.84386900	-0.46806100
O	4.03761000	-1.18161900	0.20295400
H	4.27487600	-0.27544700	-0.09330700
O	-5.46616100	2.49957300	0.71411300
H	-4.82457200	2.93111900	0.13600300
H	-6.29385700	2.97912200	0.64560400
O	-6.05500000	-2.36448000	-0.00912700
H	-6.27639200	-2.95811200	-0.72923400
H	-5.96884600	-1.47974400	-0.38640600
O	4.26849600	1.37658200	-0.57634900
H	3.30854600	1.36109200	-0.72873300
C	4.53578200	2.22650700	0.53616900
H	3.78661900	2.05863200	1.31803700

H	4.47039600	3.27333200	0.22154500
C	5.92483700	1.90988900	1.05119100
H	6.18400500	2.56212500	1.88723700
H	5.97554700	0.87318600	1.39236600
H	6.66162500	2.05093200	0.25789200
C	4.79211600	-2.12926100	-0.54616400
H	4.50757400	-3.11387300	-0.17156000
H	4.51163000	-2.07748600	-1.60427900
C	6.28263500	-1.89520500	-0.37808000
H	6.56098600	-1.96526600	0.67515900
H	6.85424100	-2.63571600	-0.94173300
H	6.55494200	-0.90182300	-0.74408900

Water-ethylene glycol (C)

C	-1.81450600	-1.16004800	0.38073100
C	-3.20645000	-1.15227400	0.42786500
C	-3.91257400	-0.05019700	-0.07662900
C	-3.20378300	1.01817700	-0.63335900
C	-1.82116700	1.01902300	-0.66784500
C	-1.13074600	-0.07998900	-0.16143600
H	-1.28458500	-2.01536800	0.77752000
H	-1.26838400	1.85217500	-1.08751300
O	-3.82520000	-2.21217300	0.97328600
H	-4.79151900	-2.20244900	0.84380500
O	-5.27399200	-0.08101400	-0.04283500
H	-5.64472300	0.79864900	0.17779800
O	-3.94375600	2.06917000	-1.13393000
H	-3.36582500	2.62397100	-1.66557800
C	0.35433800	-0.05622200	-0.22040600
O	0.92879900	-1.14301400	0.24815300
H	1.92444700	-1.08537400	0.16906100
O	0.97260000	0.89745200	-0.66624200
O	3.54282500	-1.01159200	0.01482100
H	3.74496200	-0.13659300	-0.37671900
O	-6.05263300	2.43541500	0.57257100
H	-5.46128200	2.84679600	-0.06978400
H	-6.89575500	2.89083200	0.53404300
O	-6.53080900	-2.47597100	0.31751200

H	-6.77911100	-3.14869800	-0.31940100
H	-6.49746500	-1.63480400	-0.15477400
O	3.62292300	1.61382700	-0.70581700
H	2.68282000	1.51168300	-0.93171000
C	3.68398300	2.26044300	0.55438400
H	2.94238100	1.82217800	1.23798300
H	3.45783800	3.32813400	0.45425500
C	5.07340700	2.10468400	1.13271300
H	5.13481800	2.67161000	2.06864600
H	5.81144000	2.50524500	0.43574400
C	4.20675900	-2.03323600	-0.73428000
H	4.22568800	-2.92213100	-0.10227800
H	3.63805800	-2.25482800	-1.64570500
C	5.62346800	-1.62917800	-1.10171900
H	6.04233800	-2.40207600	-1.75178200
H	5.59214600	-0.69274800	-1.67798200
O	5.42893600	0.74866200	1.34982000
H	4.67569200	0.26031800	1.70167900
O	6.47111200	-1.51353500	0.01246400
H	6.27320700	-0.68143100	0.46415100

Water-glycerol (D)

C	2.38709000	1.16707900	0.39545900
C	3.77807800	1.10508600	0.37219100
C	4.41358800	-0.05575900	-0.09460800
C	3.63620000	-1.12716100	-0.54535800
C	2.25506400	-1.07108600	-0.51383100
C	1.63483100	0.08532500	-0.04388300
H	1.91543200	2.06548000	0.76979600
H	1.65256900	-1.90683200	-0.85188200
O	4.46364100	2.16993200	0.81711100
H	5.42092500	2.11622600	0.63673900
O	5.77317900	-0.08020000	-0.12961400
H	6.12312400	-0.96482900	0.10673600
O	4.30881000	-2.23623800	-1.00921300
H	3.68302600	-2.80862100	-1.46240700
C	0.15344000	0.11381200	-0.02147500
O	-0.37747200	1.24258200	0.40395300

H	-1.38667900	1.16259100	0.40471300
O	-0.52986400	-0.83899600	-0.36700000
O	-2.93930700	1.19502200	0.36053600
H	-3.28028000	0.32505000	0.05619200
O	6.51743100	-2.58053100	0.57637300
H	5.87027200	-3.02495400	0.01571200
H	7.34223200	-3.06674500	0.52038700
O	7.12937900	2.28300100	0.01252800
H	7.37697800	2.90895900	-0.67075800
H	7.05226700	1.41763300	-0.40784000
O	-3.36559700	-1.24236200	-0.70426800
H	-2.44291300	-1.33812000	-0.97938600
C	-3.82050100	-2.45761700	-0.10472800
H	-3.95210500	-3.21054600	-0.89042200
C	-5.16663000	-2.14313000	0.52057000
H	-5.57513100	-3.04544300	0.98073000
H	-5.02358200	-1.38433400	1.30417800
C	-3.65403800	2.25056000	-0.27604600
H	-3.67928000	2.07410600	-1.35806200
C	-5.07441300	2.27945100	0.25454800
H	-5.05376800	2.45063400	1.33714300
H	-5.63450900	3.08688500	-0.23047900
C	-2.91846800	3.55242600	-0.00709500
H	-3.55137700	4.38996800	-0.30909900
H	-2.73186700	3.63676500	1.07154100
C	-2.81746000	-2.97176800	0.91648100
H	-3.22912500	-3.86021900	1.40271600
H	-2.65467000	-2.20276700	1.68337000
O	-1.72619400	3.66974300	-0.74953400
H	-1.04891200	3.09617900	-0.37660800
O	-5.64647100	1.01682200	-0.04561900
H	-6.46801400	0.89305200	0.43655100
O	-1.60040300	-3.35612200	0.31275400
H	-1.05330500	-2.57217900	0.18211200
O	-6.10343100	-1.70511700	-0.43499500
H	-5.74161400	-0.90153400	-0.82911900

Deprotonated (anionic) gallic acid

Water-methanol

C	0.25932600	-1.47672500	0.36223500
C	-1.09804100	-1.51178800	0.63837000
C	-1.84107700	-0.33486500	0.76987100
C	-1.17910300	0.88312400	0.64401000
C	0.17682800	0.92665400	0.36225300
C	0.90301300	-0.25075400	0.22024000
H	0.82657100	-2.39302800	0.25844000
H	0.66983200	1.88648500	0.25139300
O	-1.74061800	-2.71699600	0.77489000
H	-2.59646800	-2.55039300	1.18702900
O	-3.18015600	-0.47748300	1.05033500
H	-3.68852300	0.30013400	0.73908800
O	-1.91279900	2.04915900	0.80206100
H	-1.28678300	2.78003800	0.84138100
C	2.40629900	-0.19811000	-0.08244900
O	2.96479600	-1.29842400	-0.24803100
O	2.93048000	0.93887200	-0.13386600
O	5.62960700	-1.25205300	-0.90093700
H	4.66473600	-1.33611500	-0.75475400
O	-4.34067400	1.54932800	-0.33974800
H	-3.63998000	2.14296600	-0.03809000
O	-3.85063400	-2.20735600	-1.31481800
H	-3.01011000	-2.66931300	-1.23189200
H	-3.84644700	-1.61040700	-0.55703500
O	2.21247600	3.52065800	0.00798400
H	2.51353300	2.58065100	0.01448800
C	6.07930200	-0.28950700	0.02126400
H	6.23362400	-0.72213100	1.02080100
H	7.04106700	0.09959600	-0.32801800
C	-4.00862700	1.12137000	-1.65932500
H	-4.70705900	0.33313700	-1.93500200
H	-4.09737900	1.94707200	-2.37061800
H	2.66101700	3.89851800	-0.74957900
H	-2.99350700	0.71246700	-1.70126600
H	5.36852900	0.53827800	0.11184700

Water-ethanol

C	0.04764300	1.64047700	-0.49215500
C	-1.32054700	1.68694700	-0.71030400
C	-2.04975300	0.52607900	-0.97921000
C	-1.36812200	-0.68563100	-1.05017500
C	-0.00224000	-0.74293000	-0.82687000
C	0.71190900	0.41853000	-0.54830300
H	0.60689800	2.54267300	-0.27909200
H	0.50869700	-1.69881600	-0.86729500
O	-1.98895900	2.88415500	-0.64180900
H	-2.86545400	2.74947000	-1.02149500
O	-3.40556000	0.66255600	-1.16686900
H	-3.86150900	-0.11165700	-0.77378100
O	-2.10224700	-1.82844600	-1.31815700
H	-1.47748700	-2.53847800	-1.50016300
C	2.22626700	0.35480900	-0.31443300
O	2.77610800	1.43052000	-0.01272000
O	2.77025900	-0.76681700	-0.44440900
O	5.50768800	1.49815600	0.33853800
H	4.53697000	1.55778600	0.21542500
O	-4.28457000	-1.42876400	0.31192800
H	-3.60576500	-2.00221400	-0.07148700
O	-3.87607900	1.94827100	1.52860200
H	-3.06710300	2.45941700	1.42018000
H	-3.98167700	1.52434300	0.66869200
O	2.09137100	-3.31922300	-0.92145800
H	2.37130000	-2.38028100	-0.80304800
C	5.82059700	0.12428500	0.36083400
H	5.36353200	-0.39016400	-0.49247000
H	6.90990900	0.04440100	0.27058100
C	5.34836400	-0.54632200	1.64515900
H	4.26036200	-0.47745300	1.70447000
H	5.62590000	-1.60580200	1.65879000
H	5.78871200	-0.05218500	2.51554500
C	-4.08277500	-1.34497100	1.72595800
H	-4.66682100	-0.48836200	2.06441100
H	-4.49056500	-2.24836000	2.19395600
C	-2.62151900	-1.16150800	2.09368300

H	-2.51302200	-1.12728100	3.18017200
H	-2.01085800	-1.98785800	1.71691400
H	-2.23027500	-0.22936300	1.68297400
H	2.63521000	-3.80167600	-0.29754900

Water-ethylene glycol

C	0.23438400	-1.56470800	-0.74605800
C	1.54901700	-1.38602100	-1.15120600
C	2.12417200	-0.11648500	-1.23327300
C	1.33589900	0.98985700	-0.92290200
C	0.02364100	0.82671000	-0.49959100
C	-0.52636700	-0.44838800	-0.40921500
H	-0.20916900	-2.55162100	-0.68516600
H	-0.58355900	1.68539600	-0.23046500
O	2.33804800	-2.47388300	-1.41933400
H	3.17052900	-2.12753900	-1.77188900
O	3.42699900	-0.01293700	-1.65299100
H	4.00248600	-0.01885000	-0.85969600
O	1.92145500	2.22950400	-1.01731000
H	1.24854000	2.89913300	-0.85749500
C	-1.96715900	-0.62131800	0.07165100
O	-2.40228000	-1.78553700	0.13190200
O	-2.57588600	0.43463000	0.36661400
O	-5.04323900	-1.61701400	0.82838100
H	-4.08504800	-1.74180800	0.66825300
O	2.45002200	-2.48531300	1.50587100
H	1.58478100	-2.06394800	1.56605600
H	2.42243300	-2.93160900	0.64829900
O	-3.08256800	3.04379500	-0.49317600
H	-2.74103900	2.15300700	-0.31175400
C	-5.56680900	-0.83154500	-0.21402800
H	-4.79490200	-0.61762900	-0.96451000
H	-6.38321800	-1.37452500	-0.71151200
C	-6.12329100	0.47886800	0.31845200
H	-6.68185800	0.98318600	-0.48076100
H	-6.82499700	0.24746200	1.13111800
H	-3.98328700	2.95218300	-0.15930900
O	-5.12616700	1.36137900	0.78733500

H	-4.28233900	0.87415300	0.85259600
C	4.64685600	0.30202200	1.82787000
H	4.34188000	-0.09323200	2.80358700
H	5.71314800	0.54007100	1.86627700
C	3.87411000	1.56857800	1.51444800
H	2.81247100	1.32397000	1.38193500
H	3.96120500	2.26554000	2.35834400
O	4.46395900	-0.67661700	0.81669500
H	3.76623200	-1.29223100	1.10410200
O	4.40862100	2.15404600	0.34451300
H	3.68729600	2.32702700	-0.27290400

Water-glycerol

C	0.05617200	2.20322900	0.68443400
C	-1.23946700	2.69249500	0.56685200
C	-2.12860500	2.14752600	-0.36307500
C	-1.69980500	1.11498500	-1.19245400
C	-0.41328200	0.60910300	-1.07138700
C	0.45954700	1.14667800	-0.12749100
H	0.74773000	2.62786900	1.40134500
H	-0.08763100	-0.22037400	-1.69042100
O	-1.67480900	3.69798100	1.37109100
H	-2.59710100	3.85960000	1.13148300
O	-3.39984800	2.65847600	-0.40793100
H	-3.99844700	1.91417600	-0.20679800
O	-2.60976700	0.61005300	-2.08705000
H	-2.18070300	-0.08297600	-2.59922400
C	1.84204400	0.53994400	0.05730400
O	2.70463600	1.23056000	0.61319300
O	1.98063600	-0.65068400	-0.36443400
O	5.30717100	0.46187900	0.81213500
H	4.37129500	0.74357700	0.88716700
O	-1.77393200	-0.64886600	1.68403200
H	-1.12566700	-1.12463300	1.10704300
H	-1.29367500	0.11429700	2.02308800
O	-0.14246400	-2.12236200	0.14441400
H	0.68641200	-1.62522800	-0.10034300
C	5.37421300	-0.51902300	-0.20130200

H	4.59958900	-0.31692100	-0.95471900
C	5.17533500	-1.92681200	0.34583700
H	5.36014500	-2.64424600	-0.47114000
H	5.92268100	-2.10489400	1.12673000
H	0.14520100	-2.94665900	0.54360800
O	3.90934900	-2.13517100	0.91810000
H	3.24948500	-1.60841800	0.42666300
C	-5.04895600	-0.81434900	0.70409800
H	-4.98333500	-1.30124200	1.68272200
H	-6.05674300	-0.41356800	0.57743300
C	-4.77175300	-1.82127200	-0.40378000
H	-5.53424000	-2.60508100	-0.34813800
O	-4.15716600	0.28627400	0.63128900
H	-3.28073100	0.00927600	0.96639700
O	-4.89750200	-1.21064500	-1.67343300
H	-4.31424600	-0.43725900	-1.67552600
C	-3.38821800	-2.46585100	-0.27866700
H	-3.29725500	-3.24310200	-1.04056900
H	-2.61185500	-1.71904600	-0.48470100
C	6.74790300	-0.37508100	-0.84582400
H	6.81749000	-0.99004200	-1.74632900
H	7.51146400	-0.71612300	-0.13066900
O	-3.18986700	-3.07108800	0.98529900
H	-2.78438900	-2.41962300	1.56907100
O	6.98892200	0.96550700	-1.21781000
H	6.58089600	1.48589800	-0.51260500
